# Clinical research challenges in rare genetic diseases in Brazil

**DOI:** 10.1590/1678-4685-GMB-2018-0174

**Published:** 2019-06-03

**Authors:** Luciana Giugliani, Claudia Vanzella, Marina Bauer Zambrano, Karina Carvalho Donis, Thaís Klassmann Wendland Wallau, Fernando Machado da Costa, Roberto Giugliani

**Affiliations:** 1 Clinical Research Group in Medical Genetics, Hospital de Clínicas de Porto Alegre, Porto Alegre, RS, Brazil; 2 Medical Genetics Service, Hospital de Clínicas de Porto Alegre, Porto Alegre, RS, Brazil; 3 National Institute of Population Medical Genetics (INAGEMP), Porto Alegre, RS, Brazil; 4 Department of Genetics, Universidade Federal do Rio Grande do Sul (UFRGS), Porto Alegre, RS, Brazil

**Keywords:** Clinical research, clinical investigation, rare diseases, lysosomal storage diseases, enzyme replacement therapy

## Abstract

Rare diseases are defined as conditions with a prevalence of no more than 6.5 per 10,000 people. Although each rare disease individually affects a small number of people, collectively, the 6,000 to 8,000 rare conditions (80% of them with genetic cause) affect around 8% of the world’s population. Research about the natural history and underlying pathophysiological mechanisms of rare diseases, as well as clinical trials with new drugs, are important and necessary to develop new strategies for the treatment of these conditions. This report describes the experience of a clinical research group working with rare diseases in a reference center for lysosomal diseases in Brazil (Medical Genetics Service, Hospital de Clínicas de Porto Alegre). The activities of this research group enabled its participation in several international multicenter clinical research protocols related to the natural history or therapy development for rare genetic diseases. This participation has allowed the development of personal skills and institutional facilities for clinical research. The clinical research developed in our center has raised the quality of the medical assistance provided to non-clinical research patients in addition to enabling early access to new therapies to many patients with orphan conditions.

## Introduction

Rare diseases are defined as abnormal conditions that affect the health status and are infrequent (Souza *et al.*, 2010). While the definition of rare disease varies by country, prevalence-based definitions range from 1 in 500,000 to 1 in 2,000 (Haffner *et al.*, 2002; Franco, 2013). In the United States, the Orphan Drug Act of 1983 considers a disease rare based on the number of affected people. Thus, the classification of a disease as rare depends on population size (United States, 1983). In the US, a disease is considered rare if it affects fewer than 200,000 individuals, whereas other countries define a rare disease based on prevalence rates (Franco, 2013). For example, in Europe, diseases are considered rare when they affect fewer than 5 in 10,000 individuals (European Commission, 2018). In Brazil, according to the World Health Organization and Brazilian Policy for Rare Diseases (RDC 205/2017), a rare disease is defined as one affecting fewer than 65 out of 100,000 individuals (Passos-Bueno *et al.*, 2014; ANVISA, 2017). Although each disease individually affects a very small percentage of the population, collectively, the over 6,800 reported rare diseases affect up to 8% of the population and it is estimated that several million Brazilian people are affected (Federhen *et al.*, 2014).

Rare diseases are 80% genetic in nature, usually severe, chronic, and progressive with the inherent problems of limited resources, insufficient research, scarce expertise, and few patients that are geographically dispersed (Souza *et al.*, 2010; Bavisetty *et al.*, 2013). Furthermore, rare diseases constitute a major economic burden independent of a country’s size and demographics; these costs arise from increased healthcare spending and lost productivity (Dunoyer, 2011; Franco, 2013; Angelis *et al.*, 2015).

Many rare disease patients experience barriers in accessing care, and fewer than 10% receive disease-specific treatment (Melnikova, 2012). Delayed diagnoses, limited access to resources, and absence of specific therapies often preclude patients from receiving suitable, timely care. Even brief delays in diagnosis may have profound effects on outcomes; for over 40% of rare disease patients, treatment delays are precipitated by misdiagnoses (Schieppati *et al.*, 2008; EURORDIS, 2018). When patients are diagnosed, many are unable to access resources such as centers of expertise, coordinated care, patient support systems, and effective treatment. For many rare diseases, there are no effective treatments and information on disease progression is limited. Therefore, research into the natural history and underlying pathophysiological mechanisms of rare diseases and clinical trials are necessary to develop a foundation for discovering targeted medicines (Dharssi *et al.*, 2017).

Clinical research on rare diseases faces many challenges when conducting trials in small populations. Standard trial designs are not optimized to obtain adequate safety and efficacy data from small number of patients, so alternative designs need to be considered (Gupta *et al.*, 2011; Abrahamyan *et al.*, 2016). Affected patients can be hard to identify, especially in the early stages of disease, are generally geographically dispersed, and are often children. Trials are frequently conducted on an international scale, might be subject to complex and multiple regulatory agency oversights, and affected by local customs, cultures, and practices (Kempf *et al.*, 2018).

This review aims to describe the experience of a clinical research group working with rare diseases in a reference center for lysosomal diseases. In the following sections, we will briefly describe the most relevant activities related to clinical research in our center.

## Institutional infrastructure for clinical research

The Hospital de Clínicas de Porto Alegre (HCPA), located in the capital city (Porto Alegre) of the southernmost state of Brazil (Rio Grande do Sul), has a Medical Genetics Service (MGS-HCPA). MGS-HCP is a well-known reference center for rare genetic diseases in the continent, and has been recognized since 2004 as a WHO Collaborating Center (WHO-CC) for the Development of Medical Genetic Services in Latin America. This comprehensive genetic service includes several laboratories and hosts several networks in the field of inborn errors of metabolism (such as the MPS Brazil Network, IEM Brazil Network, the NPC Brazil Network, and the LSD Brazil Network). Additionally, the center serves as a place for diagnostic support and data collection for these rare conditions, as well as for research and education in this area (Giugliani *et al.*, 2016). We will focus on the clinical research group, which participates in several national and international clinical research protocols related to the natural history of, or therapy development for lysosomal diseases.

Our Clinical Research Group in Medical Genetics (CRGMG) works in a facility specifically built for clinical research activities - The Clinical Research Center at Hospital de Clínicas de Porto Alegre (CRC/HCPA). The CRC/HCPA was inaugurated in 2009 with the main objective of promoting the development and qualification of clinical studies carried out in our institution and contains all necessary infrastructure for the development of all stages of clinical and epidemiological studies.

In the CRC/HCPA, several studies are ongoing in different areas such as genetics, cardiology, dermatology, endocrinology, psychiatry, gastroenterology, hematology, gynecology, infectious disease, mastology, nephrology, neurology, nutrition, rheumatology, ophthalmology, oncology, pneumology, and urology. All these groups are committed with clinical research and human resource training.

The CRC/HCPA has several facilities, such as outpatient clinics, with access to electronic medical records, temperature-controlled room with an energy generator for medication storage, an area for the collection and processing of biological samples, and many shared facilities for meetings, seminars, monitoring, and video conferences. In addition, there is a ward with facilities for infusions of pediatric and adult patients, with a dedicated nursing team and emergency chart. Each clinical research group has private offices with space for study coordinators, sub-investigators, data entry person, and logistic assistants. Cabinets for the short- and long-term storage of study documents are also available.

The CRC/HCPA is connected to the HCPA main building, where many study procedures such as imaging and functional tests are performed, and where study patients can be admitted when hospitalization is required.

## Team organization and qualifications

The staff is one of the most important components of a clinical research team; their qualifications and experience are essential to the success of clinical protocols (Baer *et al.*, 2011). The CRGMG currently has a permanent staff of more than 20 people, which includes principal investigator (PI), sub-investigators, study coordinators, infusion nurses, pharmacists, and logistic and administrative support and data entry assistants. In addition, this research group has many other collaborators according to the specialties required for each clinical study.

## Before, during, and after protocol regulatory submission

Regulatory submissions are the most critical milestones in our clinical research program. In Brazil, study protocols should be evaluated and approved by the local ethics committee and, in certain cases (as when there is an international sponsor), also by a National Commission for Research Education (CONEP). For projects involving the development of new therapies, protocols must also be evaluated and approved by the Brazilian Sanitary Vigilance Agency (Agência Nacional de Vigilância Sanitária – ANVISA). When a study involves a genetically modified product, it must also be reviewed by local and national biosafety committees.

Regulatory documents are submitted to track and evaluate the ethical and procedural conduct of a trial and the quality of the data that is produced. In addition, the regulatory dossier demonstrates the compliance of the investigator, sponsor, and ethics committees (IRB/IEC) with the standards of the International Confederation for Harmonization (Integrated Addendum to ICH, 2016); the Brazilian Regulatory Agency (ANVISA) is a member of GCP-ICH since November 2016.

Since early 2012, all clinical trial application materials are submitted through Brazil’s national and unified electronic database called [Bibr B34]“Plataforma Brasil” (http://plataformabrasil.saude.gov.br/login.jsf). The implementation of the “Plataforma Brasil” system, along with several other measures (as the increase of ad-hoc consultants), has reduced the regulatory review time.

**Figure 1:**
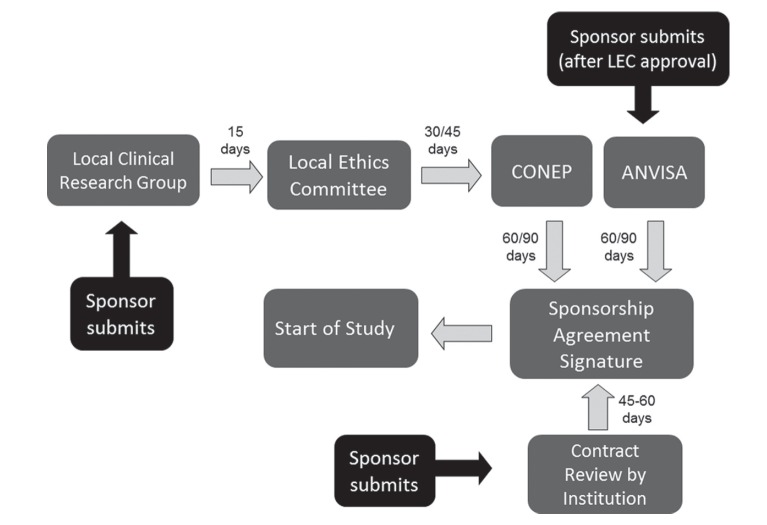
Regulatory submission steps for our clinical research group in Brazil.

In the first step, the sponsor sends the regulatory dossier to our CRGMG, which reviews the documents and submit them to “Plataforma Brasil”. The regulatory dossier (including the informed consent form) must be in accordance to the Resolution 466/2012 and their complementary norms for CONEP, and RDC 09/2015 for drugs for ANVISA. Next, the local ethics reviews the protocol. After approval, the protocol can be sent to CONEP (when necessary, such as when there is an international sponsor) and to ANVISA (when the protocol involves a new therapy or importation of devices and/or collection kits). The overall regulatory approval (Figure 1) may take 3 to 6 months (the time was recently reduced as fast-track processes were implemented for protocols involving rare diseases, both by CONEP and ANVISA).

Following regulatory approval, a site initiation visit is scheduled and the study can begin enrolling patients. Throughout the study, all serious adverse events must be reported to the ethics committee, and study reports (every 6 months), new amendments, notifications, and protocol deviations must be sent.

All documents related to the regulatory dossier, as well as study documents, must be filed in the study binder. After completion of the study, all documents will be stored at the site for 15 years or as directed by the sponsor.

## Recruitment and support of patients

The successful recruitment and retention of patients is challenging in clinical research. All members of a research team, as well as trial sponsors, need to be involved in a collaborative recruitment effort. The successful recruitment of participants depends on factors such as administrative support, clinical staff expertise, volume/turnover of patients, realistic study protocols, and stability of the patient population (Kadam *et al.*, 2016).

Our CRGMG has the objective of recruiting individuals who may be eligible to enter a research protocol in accordance with the international standards of good clinical practice. The principal investigator, sub-investigators, and study coordinators can recruit patients at our site. Our team follows an internal patient recruitment protocol, which includes searching for potential candidates that might meet inclusion criteria in patient databases, contacting the assistant physicians to explain the study design and invite them to refer patients for participation in the clinical research. Thereafter, we contact the patient directly or via the physician’s assistant to check the patient’s interest and availability in participating in the study. If the patient agrees, a visit to the CRC/HCPA is scheduled to provide more detailed information about the project and obtain a signed informed consent form to start the screening process.

Our group works closely with patients’ physicians to keep them informed about the selection process, communicate patients’ decision to participate or not in the study, as well as any relevant events that concern patients’ health that require care during the course of the study. After the participation in the study is completed, our group refers the patient to the physician, along with a medical report and a copy of the tests performed. Furthermore, our group provides administrative and logistical support for patients and their companions to participate in the study throughout the entire protocol. In addition, if patients experience any side effects during the study, the sponsor is notified and a medical team is readily available to minimize symptoms; our group can also be supported by HCPA.

For successful recruitment, enrollment, and support of patients in the clinical research protocol, every member of the research team needs to be motivated, have good interpersonal skills, and establish a trusting relationship with the family, in addition to provide adequate, clear, and concise explanations about trial procedures (Ruckmani *et al.*, 2012).

## Patient reported outcome measures (PROMs)

Rare diseases can impact a patient’s daily activities and individuals might be dependent on family or caregivers to manage their everyday needs. This loss of autonomy can have a negative impact on their quality of life (QoL) and affect family and caregivers given the burden of care and the young age of many patinets. As a result, families have reported psychosocial concerns and feelings of isolation and depression (Slade *et al.*, 2018).

Therefore, rare diseases can lead to a significant reduction in quality of life for patients and their families. The patients’ voice must be central to clinical decision for an effective deliver, evaluation, and understanding of therapeutic interventions. Patient reported outcome measures (PROMs) are used to capture patients’ views about their health status and facilitate our understanding of the impact of these diseases and their treatments on patient’s quality of life and symptoms (Slade *et al.*, 2018).

The European Medical Agency (EMA, 2014, 2016) and the Food and Drug Administration (FDA) have recognized the importance of including the patient’s perspective in development of PROMs, and the FDA guidelines state that patient input is a requirement for applications using PROMs to support clinical trial and medical labeling claims (FDA, 2009). It has also been demonstrated that PROMs developed using the patient’s perspective are more robust and provide more sensitive and specific measurements.

Most of the rare diseases lack disease-specific PROMs that can be used to gain a better understanding of the issues that patients experience. For PROMs to be effective in clinical practice, they have to capture the disease characteristics that matter to the patient. Selection of suitable PROMs should reflect these properties and domains as well as the disease natural history and prognosis. This can be challenging because of the heterogeneity of patients’ experiences and disease presentations caused by cultural influences, and genetic, and phenotype variations (Trujillano *et al.*, 2017).

The importance of ensuring that the patients voice is central to clinical decision making is key to delivering, evaluating and understanding therapeutic interventions. The dearth of valid PROMs for use in many rare conditions and difficulties in validating currently available PROMs makes evaluating effective treatments problematic. Patient input throughout the development of PROMs including qualitative research is essential to ensure that outcomes that matter to people living with rare disease are appropriately captured. Use of PROMs in rare disease research and clinical practice offers the potential to improve patient care and outcomes, as well as the applicability of study result (Slade *et al.*, 2018).

## Contract and project management

Clinical trials involve a large number of tasks and require people responsible for each part of the overall study. The relationship between these parties requires understanding and careful management for a successful study. The project management and logistical support for participants are key points in clinical research (Snyder *et al.*, 2016).

Each research protocol has an individual contract, signed by the investigator, sponsor, institution and, if needed, by an administrative partner. Each party with its respective responsibility should always act in accordance with good clinical practice ([Bibr B27] Red, PARF 2005), the protocol, and all applicable local regulations governing the conduct of clinical trials.

Throughout the study protocol, our group provides logistical support for patients and their families, such as purchasing bus/flight tickets, transfer to the center, hotel accommodation, meals, and other expenses. Our team also closely follows any adverse event that patients present throughout the study, to ensure patient safety and integrity.

## Types of study: Our experience

The methodology chosen for research is very important to the proper development of the study and minimization of bias. The CRGMG has extensive experience in conducting retrospective, observational, natural history, exploratory biomarker, phase I, II, III, and IV studies, and extension studies.

**Figure 2:**
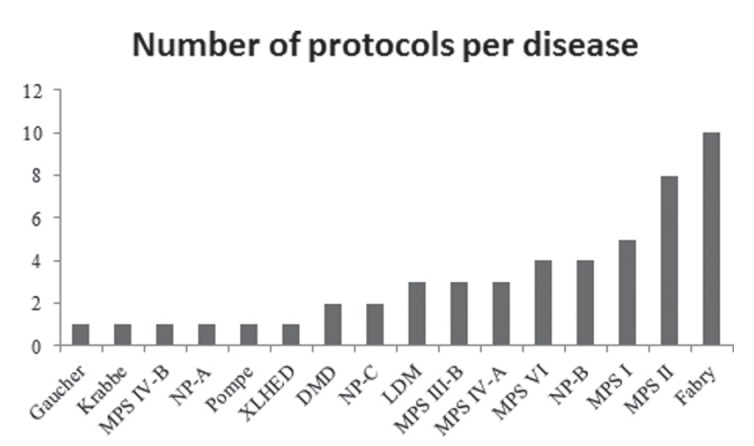
Number of persons in the Clinical Research group in Medical Genetics per disease.

As mentioned previously, our group began its activities in 2000, and to-date, more than 50 clinical research protocols have been conducted in the field of lysosomal storage diseases (LSDs). Figure 2 illustrates the number distribution of protocols per disease. We highlight several studies involving mucopolysaccharidoses (MPS) and Fabry Disease, among other LSDs. To-date, approximately 500 patients have been included in clinical studies developed by our team, most of which with MPS type II, MPS IVA, and Fabry Disease.

**Figure 3:**
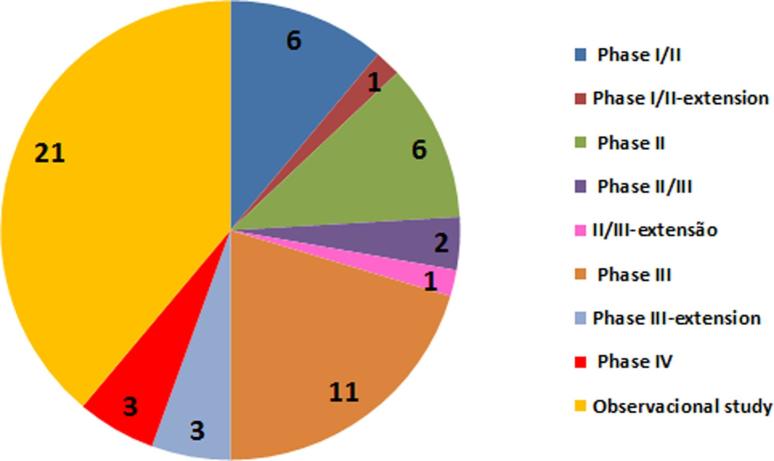
Clinical study phases for the Clinical Research group in Medical Genetics.

Although most of our studies were observational, the proportion of clinical trials involving new drugs has been increasing due to the emergence of new therapeutic approaches for rare diseases (Figure 3). The goal of most studies is to not only test new therapies but also evaluate long-term therapies previously approved, compare different treatments, identify biomarkers, and evaluate the natural history of the disease, which are issues of great relevance to rare and complex diseases (Federhen *et al.*, 2014). The clinical studies registry by period in the Clinical Trials Database (www.clinicaltrials.gov) shows that there has been an increase in the number of clinical research protocols since the beginning of this century.

The CRGMG has participated in several trials of drugs that are currently approved, such as for MPS VI, MPS II, MPS IVA, and Fabry disease, being in many cases the top enrolling center. The infrastructure available and the team expertise, along with the number of potential study subjects, has attracted many companies to perform clinical trials at our center, providing to hundreds of Brazilian patients early access to innovative experimental therapies for rare orphan diseases.

## Monitoring and auditing

The quality and integrity of clinical trials and associated data are derived not only from accurate trial results but are also closely related to the authenticity and integrity of the data and documents and procedural compliance during data collection. Compliance with GCP and standards indicates the reliability, completeness, and accuracy of data and documents, which is essential to demonstrate drug efficacy and safety in clinical trials. Therefore, the monitoring and auditing of clinical trials and associated data helps to verify the reliability and accuracy of the data and validates compliance with GCP, which is an international ethical and scientific quality standard for the design, conduct, performance, monitoring, auditing, recording, analyses, and reporting of clinical trials. It also serves to protect the rights, integrity, and confidentiality of trial subjects (Liu *et al.*, 2015).

Investigators must submit protocols to a research ethics committee for review and approval prior to initiation of a study. The research ethics committee performs an initial and continuing review of the study until completion. Continuing review is one of the major challenges of research ethics committees, as it involves a number of aspects including annual renewals, amendments to the approved protocols, serious adverse event monitoring, on-site monitoring, and auditing (Ochieng *et al.*, 2013). Monitoring and auditing of clinical trials is necessary to ensure research conduct compliance.

Monitoring is defined as “the act of overseeing the progress of a clinical trial, and ensuring that it is conducted, recorded, and reported in accordance with the protocol, standard operating procedures (SOPs), GCP-ICH, and the applicable regulatory requirement(s)” (Integrated Addendum to ICH, 2016). Monitoring is a quality control function where study conduct is routinely assessed on an ongoing basis throughout the trial. On-site monitoring of research is one of the most effective ways to ensure compliance. Monitoring visits often occur on-site as requested by the sponsor, and the monitoring teams generally evaluate the following items: regulatory issues, site facilities, informed consent process and documentation, participant welfare, reporting and management of adverse events, study-related training, and working practices, which must comply with a Normative Instruction (IN.20,02/10/2017) about Good Clinical Practice inspection procedures on Clinical Trials published by ANVISA and GCP-ICH regulations (Ochieng *et al.*, 2013).

Auditing is defined as “a systematic and independent examination of trial-related activities and documents to determine whether the evaluated trial-related activities were conducted, and the data recorded, analyzed, and accurately reported according to the protocol, sponsor’s SOPs, GCP, and the applicable regulatory requirement(s)” (Integrated Addendum to ICH, 2016). Auditing, a quality assurance function, is an independent, top-down, systematic evaluation of trial processes and quality control. Audit teams can assess a wider study sample than monitor teams and can help evaluate trends at various levels by auditing a single site or multiple sites, trial vendors and/or the sponsor. Auditors may look at study design, site/data management, statistical analysis, and the clinical study report. In general, auditors evaluate compliance to recognized standards (i.e., the FDA’s Code of Federal Regulations, International Conference on Harmonization, International Standards Organization, and SOPs).

Our monitoring and auditing processes have demonstrated that we have adequate facilities to conduct studies, and they were all compliant with GCP and ethical standards. Furthermore, we have received several sponsors audits, which reported minor and easily resolved findings. Two inspections by the FDA identified no issues and concluded that our service adheres to the applicable statutory requirements and FDA regulations governing the conduct of clinical investigations and the protection of human subjects.

## Clinical research: Challenges and benefits

The Brazil participation in clinical trials for a new rare disease therapy opens doors to research participant, with the possibility of changing the natural progress of the disease. In recent years, public-private partnerships between academic institutions and pharmaceutical companies have grown in our country (Interfarma, 2013; Federhen *et al.*, 2014). Our group has participated in several international multicenter clinical trials (phases I, II, III, and IV) for the development of new therapies for many different lysosomal diseases, including Fabry, Pompe, Metachromatic Leucodystrophy, MPS I, MPS II, MPS IVA, MPS VI, and others.

One of the benefits of conducting clinical research in Brazil is to provide patients with an orphan disease an opportunity to access experimental therapies. In addition, the high standards of the research protocol are transferred to clinical practice, making professionals more discerning and demanding, and improving the quality of care.

In 2014, the Ministry of Health introduced the “Policy for the Integral Attention to Subjects with Rare Diseases” in Brazil. The policy established guidelines for offering comprehensive care (diagnosis, treatment and/or long-term management) to individuals affected by rare diseases in the public unified health system (Passos-Bueno *et al.*, 2014; Giugliani *et al.*, 2016). The regulatory requirements for clinical trials are important and are intended to ensure the protection of research participants, regardless of the disease. However, such requirements should be considered differently for rare diseases. Thus, new resolutions for rare disease have been approved and published in recent years. Among these, one resolution issued by the National Ethics Committee exempts ultra-rare diseases from the requirement of life-long post-trial supply of the experimental drug. Also, the RDC 205/2017, issued by ANVISA in December 2017, establishes a special procedure for the approval of clinical trials and registration of investigational products for the treatment, diagnosis, or prevention of rare diseases.

Working with rare diseases is a challenge in an emerging country with health and research systems still under development. To overcome these challenges, it is necessary to establish partnerships involving health authorities, research centers, professionals and researchers, patients, family members, and pharmaceutical companies. The adequate development of innovative research provides patients access to treatments, qualifies the professionals and research institutions (with repercussions for the assistance activity), and project the country into the international arena.

In conclusion, the activities of the CRGMG enable Brazil to participate in several international multicenter clinical research protocols related to the natural history or therapy development for rare genetic diseases. This participation has allowed many Brazilian groups to develop skills and facilities for clinical research, which subsequently enabled some centers to develop original clinical research projects. In addition, we believe that the development of clinical research contributes to the growth and qualification of the Medical Genetics Service of the Hospital de Clínicas de Porte Alegre, bringing results and new therapeutic possibilities, and raising the awareness of these diseases among health professionals, families, and the community.
